# Unexpected Motherhood-Triggered Hearing Loss in the Two-Pore Channel (TPC) Mutant Mouse

**DOI:** 10.3390/biomedicines10071708

**Published:** 2022-07-15

**Authors:** Juliette Royer, José-Manuel Cancela, Jean-Marc Edeline

**Affiliations:** Institut des Neurosciences Paris-Saclay (NeuroPSI), Université Paris-Saclay, CNRS, Centre CEA Paris-Saclay–bât 151, 91400 Saclay, France; juliette.royer@cnrs.fr (J.R.); jose-manuel.cancela@universite-paris-saclay.fr (J.-M.C.)

**Keywords:** auditory brainstem response, auditory threshold, calcium regulation, hormonal factors, parturition

## Abstract

Calcium signaling is crucial for many physiological processes and can mobilize intracellular calcium stores in response to environmental sensory stimuli. The endolysosomal two-pore channel (TPC), regulated by the second messenger nicotinic acid adenine dinucleotide phosphate (NAADP), is one of the key components in calcium signaling. However, its role in neuronal physiology remains largely unknown. Here, we investigated to what extent the acoustic thresholds differed between the WT mice and the TPC KO mice. We determined the thresholds based on the auditory brainstem responses (ABRs) at five frequencies (between 4 and 32 kHz) and found no threshold difference between the WT and KO in virgin female mice. Surprisingly, in lactating mothers (at P9–P10), the thresholds were higher from 8 to 32 kHz in the TPC KO mice compared to the WT mice. This result indicates that in the TPC KO mice, physiological events occurring during parturition altered the detection of sounds already at the brainstem level, or even earlier.

## 1. Introduction

Hearing loss, the most common sensory impairment in humans, affects a growing proportion of people all over the world. Hearing loss can be congenital or acquired and can be caused by many genetic and/or environmental factors. About half of these disorders have a genetic basis, with hearing loss occurring either as an isolated condition (non-syndromic) or with additional phenotypes (syndromic). Over the last three decades, about 110 genes responsible for non-syndromic deafness and more than 300 genes responsible for syndromic forms of deafness in humans and/or mice have been reported [1,2]. However, many genes remain to be identified, in particular those responsible for rare forms of deafness [3,4,5], most often syndromic, or those involved in the most common form of deafness in adults, namely age-related hearing loss (ARHL) or presbycusis [6,7,8]. ARHL is considered to result from interactions between environmental factors (e.g., infections, noise exposure, ototoxic drugs) and hereditary factors [9,10]. Although some of these genes have a well-established causal role in ARHL, the identification of other genes, or the associations between them inducing hearing loss, have not been fully investigated.

Over these last decades, several environmental factors have been found to influence the expression of ARHL. A first obvious factor is noise exposure (acute or chronic), which can exacerbate ARHL. In a set of elegant studies, Kujawa and Liberman [11,12] demonstrated that a single 2 h exposure at 100 dB performed in young animals (4 month old mice) is enough to trigger an accelerated aging in older animals (1.8-year-old mice). Such a single noise exposure can cause temporary, but totally reversible, threshold elevation. It leaves cochlear sensory cells intact but causes acute loss of afferent nerve terminals and delayed degeneration of the cochlear nerve [11,12].

The status of the stria vascularis (SV) and its vasculature has been measured in aged human temporal bones and represents a major pathology of hearing loss [13,14]. The functions of the SV are to generate the endocochlear potential (EP), regulate the secretion of the endolymph, and maintain cochlear homeostasis. In gerbil models, it has been shown that SV degeneration correlates with reduced Na + K + ATPase and Na-K-Cl cotransporter1 (NKCC1) expression, required for K+ homeostasis to produce the EP [15,16,17,18]. In these studies, a declining value of the EP has also been measured, supporting a strial presbycusis phenotype of ARHL [19,20].

Another cause of ARHL occurring at the periphery is the sclerosis of the middle ear bones. Otosclerosis is a disease of the otic capsule bone responsible for the abnormal growth of poor-quality new bone in the middle ear. This new bone blocks the stape movements, causing conductive hearing loss. This disease, which affects 0.2 to 1% of the population, mostly occurs between 20 and 40 years old, and is more prevalent in women, suggesting that hormonal factors are involved. Half of the cases of otosclerosis are so-called familial forms, indicating genetic transmission. However, no specific gene has been identified yet, and the mode of hereditary transmission would be autosomal dominant. The other half of the cases constitute sporadic forms. For women, it is not uncommon for otosclerosis to appear, or worsen, during pregnancy, which explains why otosclerosis has been initially associated with pregnancy [21]. This link is still debated today, because of the paucity and the heterogeneity of the results, and the difficulty in isolating a single genetic factor [22,23].

Numerous studies have shown that lysosomes and autophagy dysfunction underlying ARHL are often associated with cochlear hair cell degeneration [24,25,26]. For example, it has been shown that the double deletion of TRPML1 and TRPML3, two endo-lysosomal calcium channels, diminish outer hair cell longevity and accelerate ARHL [24]. Intriguingly, endo-lysosomes express additional calcium channels [27], namely the two-pore channels (TPC) [28,29,30], the role of which is poorly understood in neuronal physiology [27]. Two TPC isoforms are expressed in rodents and in human. The TPC1 isoform is mostly present in endosomes, whereas the TPC2 isoform is mostly lysosomal. Both TPCs have been shown to carry Ca^2+^ although they also display H^+^ and Na^+^ conductances [31,32]. TPCs have been involved in autophagy regulation and their deletion in various cell types has led to impaired autophagy fluxes [33,34,35]. Importantly, TPCs have also been shown to regulate hormone and enzyme secretion [28,36] as well as being recruited by glutamate to modulate neuronal excitability [37] and by VEGF2 to control neoangiogenesis [38].

To better understand the link between lysosomal Ca^2+^ stores and neuronal physiology, a mouse lacking endolysosomal two-pore channels (TPC KO) was created, knowing that the copy number variation of genes encoding TPCNs in chromosomal regions in humans have been associated with autism spectrum disorders (ASD), a disorder often associated with sensory perception impairments. In these TPC KO mice, altered social behaviors have recently been reported (Martucci et al., submitted).

Based upon auditory brainstem responses (ABRs), we report here the surprising discovery, that despite similar audiograms in normal physiological conditions (virgin mice), at 9–10 days post-partum, TPC KO lactating mothers displayed higher ABR thresholds compared to the WT mothers. This sudden increase in the ABR threshold observed in our study could potentially be related to the rare disease of sudden sensorineural hearing loss reported in pregnant women.

## 2. Materials and Methods

### 2.1. Animals

These experiments were performed under the national license A-91-557 (project 12471) using procedure No. 32-2011 validated by the Ethics Committee No. 59 (CEEA Paris Centre et Sud). All procedures were performed in accordance with the guidelines established by the European Communities Council Directive (2010/63/EU Council Directive Decree). Data were from four groups of animals. First, we used 15 virgin and 15 mother WT mice (9 to 24 weeks old) weighing from 21 to 40 g (virgins median: 25 g; mothers median: 33 g). Second, we used 14 virgin and 19 mother TPC KO mice (10 to 25 weeks old) weighing from 22 g to 37 g (virgin median 25 g; mother median 31 g). Globally, there was no age difference between the two genotypes (*t*-test < 1, ns). The TPC KO (TPC1/2^−/−^) mice were generated by crossing mutants homozygous for the TPC1 isoform (Tpcn1^T159^) [39] obtained by the European Mouse Mutant Archive (EMMA) and mutants homozygous for the TPC2 isoform (Tpcn2^YHD437^) [28]. The TPC KO mice and WT mice came from our own colony housed in a humidity (50–55%) and temperature (22–24 °C)-controlled facility on a 12 h/12 h light/dark cycle (light on at 7:30 A.M.) with free access to food and water. All mice were genotyped as previously described [39]. On the day of the experiment, mother mice (WT and TPC KO) were lactating with at least four pups aged 9–10 days old.

### 2.2. Pure-Tone Audiogram

Pure-tone audiograms were determined by testing the auditory brainstem responses (ABRs) under deep anesthesia (95 mg/kg ketamine, 24 mg/kg xylazine, i.p.). Mice were placed on a heating blanket to avoid hypothermia. Auditory stimuli (20 ms in duration, rise-fall time 2 ms) were presented monaurally using an insert earphone (Knowles Electronics) placed in the animal right ear. ABRs were recorded using two subcutaneous electrodes (SC25; Neuroservice, Aix-en-Provence, France) located just above the tympanic bulla and skull dorsal midline, and one ground electrode was placed in the thigh. Insertion of the subcutaneous electrodes was not associated with any sign of discomfort. The signal was digitized (sampling rate 32,000 Hz) and filtered (150–1500 Hz). The software (RTLab, Echodia, Clermont-Ferrand, France) allowed for the averaging of 500 responses during the presentation of five pure-tone frequencies (between 4 and 32 kHz). The auditory threshold for each frequency was the lowest intensity at which a detectable ABR wave could still be detected (usually waves II and III). For each frequency, the threshold was determined by gradually decreasing the sound intensity (from 70 dB down to −10 dB SPL). For the four groups, we excluded partially deaf animals (i.e., animals exhibiting a click threshold ≥70 dB SPL). This was the case for 3/33 WT mice (10%) and for 5/38 KO mice (13%). Aside from these animals, all the animals used in this study had normal pure-tone audiograms for their age (9–25 weeks) and for the C57 BL/6 genetic background [40,41,42,43]. During the off-line analyses, the amplitudes and latencies of the five ABR waves were quantified at 70 dB SLP for the four groups.

### 2.3. Statistics

All data are presented as mean values ± standard error (s.e.m.). To assess the significance of between group differences for the ABR thresholds, we used unpaired *t*-tests. To compare the mean amplitude and latency of the five ABR waves at 16 or 8 kHz between groups, a N-way ANOVA was performed. Post-hoc comparisons were performed with Fisher LSD tests, which were corrected for multiple comparisons using Bonferroni corrections and were considered as significant if the p value was below 0.05. In all figures displaying group data, we indicated by * a *p* value < 0.05.

## 3. Results

**Figure 1:**
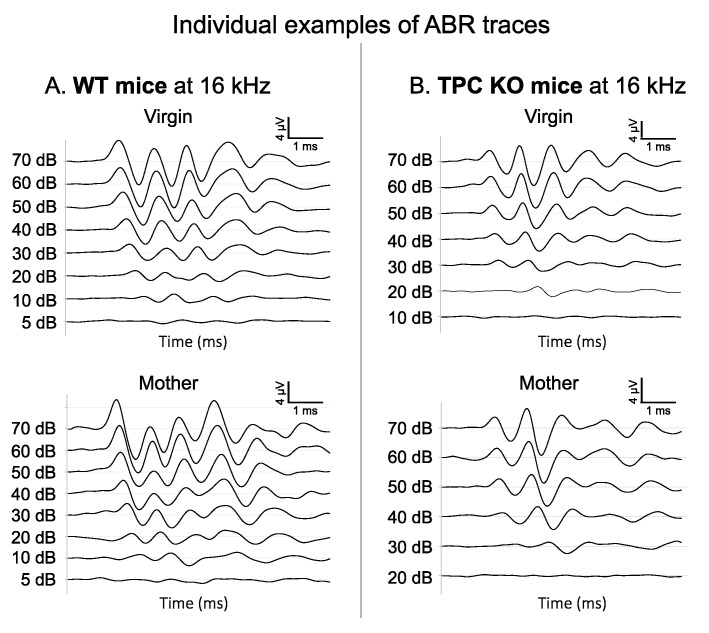
Individual examples of the ABR traces for the four groups. (**A**) Example of an auditory brainstem response (ABR) of a WT virgin mouse (top) and a WT mother mouse (bottom) when a 16 kHz pure tone is presented at different intensities (between 70 and −10 dB SPL). The threshold of the virgin mouse was at 10 dB SPL and the one of the mother mouse was at 10 dB SPL. (**B**) Example of the auditory brainstem response (ABR) of a TPC KO virgin mouse (top) and a TPC KO mother mouse (bottom) when a 16 kHz pure tone is presented at different intensities (between 70 and −10 dB SPL). The threshold of the virgin mouse was at 20 dB SPL and the one of the mother mouse was at 30 dB SPL. Mother TPC KO mice displayed higher thresholds than the mother WT mice, which was not the case in the virgin mice.

Figure 1 displays the individual examples of the average ABR traces obtained at 16 kHz from 70 dB to sub-threshold intensities. Each trace represents the average responses to 500 stimulus presentations. As shown in Figure 1A, the ABR threshold of the WT (virgin and mother) mice was at 10 dB: at 5 dB, the most prominent waves (usually waves II and III) could no longer be detected. Figure 1B shows the ABR waves of a virgin TPC KO mouse (top) where waves were still detectable at 20 dB. In contrast, these waves were not detected at 20 dB for a mother TPC KO mouse (bottom) and the threshold was at 30 dB, higher than the three other mice.

**Figure 2:**
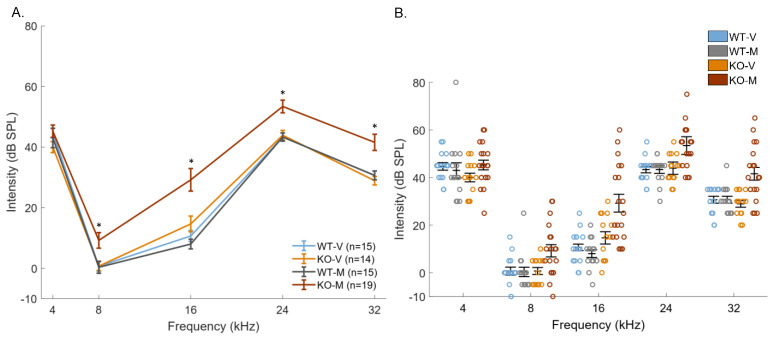
The mean ABR thresholds for the four groups. (**A**) Pure tones (at 4, 8, 16, 24, and 32 kHz) were presented at different intensities (between 70 and −10 dB SPL) and the auditory brainstem response (ABR) thresholds were determined for each frequency (wave III threshold measurement). Each point represents the average threshold (±s.e.m.) of each group at different frequencies. The ABR thresholds measured in WT virgins (blue), TPC KO virgins (orange), and WT mothers (gray) mice, were not different between groups. Only the thresholds of the TPC KO mother mice (brown) were significantly higher at 8, 16, 24, and 32 kHz than the other groups. (**B**) The individual ABR thresholds at 4, 8, 16, 24, and 32 kHz in each group are represented by circles. The average threshold and standard error are represented by horizontal markers. The * indicate significant difference (*p* < 0.05) between TPC KO mothers and WT mothers.

Figure 2 presents the mean ABR thresholds for the four groups at the five tested frequencies (4, 8, 16, 24, and 32 kHz). A N-way ANOVA compared the thresholds in the four groups. There was a significant group-effect (F_3,4_ = 30.26, *p* < 0.001), a significant frequency-effect (F_3,4_ = 280.77, *p* < 0.001), and a significant interaction between the groups and frequencies (F_3,4_ = 178.5, *p* < 0.01). At all of the tested frequencies, the mean ABR threshold did not differ between the WT virgin and WT mother mice (unpaired *t*-tests, all *p*-values > 0.05). The mean ABR threshold of the virgin TPC KO mice did not differ from that of the WT (virgin and mother) mice at 4, 8, 24, and 32 kHz (unpaired *t*-tests, all *p*-values > 0.05). In the virgin TPC KO mice, it was only at 16 kHz that the mean threshold was slightly higher, and only compared to the WT mother mice (unpaired *t*-tests, *p* = 0.0356). In contrast, in the mother TPC KO mice, the mean ABR thresholds was significantly higher at 8, 16, 24, and 32 kHz compared to the three other groups (Figure 2A; all unpaired *t*-tests, *p* < 0.005 at 8, 16, 24, and 32 kHz).

Figure 2B reveals that, except for the TPC KO mother mice, the individual ABR thresholds were rather homogeneous. It appears that the increased thresholds at 8, 16, 24, and 32 kHz in the TPC KO mother mice were caused by a subgroup of mice (n = 9), which displayed a threshold out of the range of the other groups, while the other TPC KO mother mice had normal ABR thresholds. Thus, the increase in thresholds did not emerge in all of the TPC KO mother mice. This increase in threshold could potentially be the consequence that the mothers were older than the virgins in this study. However, this was the reverse: the TPC KO mothers were younger than the TPC KO virgins (unpaired *t*-test, *p* < 0.05). In addition, there was no relationship between the threshold and age in the TPC KO mice at all of the tested frequencies. Supplementary Figure S1 shows this lack of relationship at both 8 kHz (R = −0.08, *p* = 0.64) and 16 kHz (R = −0.18, *p* = 0.31).

**Figure 3:**
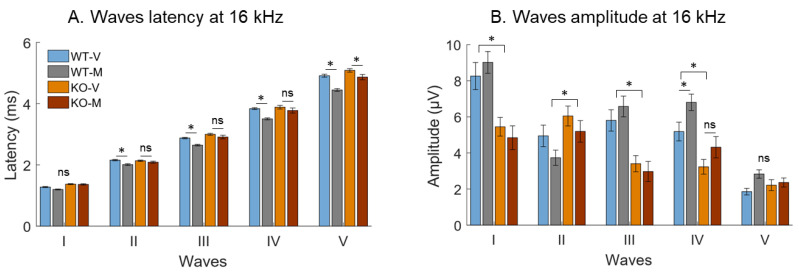
The mean latency and mean amplitude of the five ABR waves at 16 kHz at 70 dB. (**A**) Latency distribution of waves I to V for each group at 16 kHz. The latencies of waves II to V were significantly shorter in the WT mothers compared to the WT virgins. Wave latency in the TPC KO mother mice was only shorter at wave V compared to the TPC KO virgins. (**B**) The amplitude distribution of waves I to V for each group at 16 kHz. The amplitudes of waves were not different between the virgin and mother mice (WT and TPC KO), except for wave IV between the WT virgins and WT mothers. The wave amplitude between the WT and TPC KO mice also differed for four of the five ABR waves. The * indicate significant difference (*p* < 0.05) and ns indicate non-significant effects.

To go further, we quantified the latency and the amplitude of the ABR waves in virgins and in mothers at 8 and 16 kHz as this frequency range often shows lower thresholds in many mouse strains [44]. We present here only the mean latency (Figure 3A) and mean amplitude (Figure 3B) for the five ABR waves at 16 kHz for the highest intensity tested (70 dB). A N-way ANOVA compared the amplitude or the latency of the five ABR waves in mothers and in virgins. In the WT mice, this analysis revealed that the latencies of waves II to V were significantly and systematically shorter in mothers compared to virgins (Figure 3A, all unpaired *t*-tests *p* < 0.05). There was a significant group-effect (F_1.14_ = 165.89, all *p*-value *p* < 0.0001), a wave-effect (all *p*-values *p* < 0.0001), and a tendency for interaction between the groups and waves (*p* = 0.08). For the TPC KO mice, only the latency of wave V was found to be shorter in the mothers compared to virgins (Figure 3A, unpaired *t*-tests *p* <0.05). There was a significant group-effect (F_1.18_ = 6.73, all *p*-value *p* < 0.05) and a wave-effect (all *p*-values < 0.0001), but there was no interaction between the groups and waves (*p* = 0.44).

The analysis of the amplitude of the five ABR waves in the WT mice indicated that there was a tendency for a group-effect (Figure 3B, F_1.14_ = 3.15, *p* = 0.07). The wave-effect was systematically significant (all *p*-values < 0.0001) and there was no interaction between the groups and waves (lower *p*-value: *p* = 0.0855). Post-hoc comparisons revealed that the mean amplitude of wave IV was higher in the mothers than in the virgins (unpaired *t*-tests *p* <0.05). For the TPC KO mice, there was no group-effect (Figure 3B, lower *p*-value *p* = 0.6944), but there was a significant wave-effect (all *p*-values *p* < 0.0001) and there was no interaction between the groups and waves (lower *p*-value: *p* = 0.3775). Post-hoc analysis did not reveal significant differences between the virgin and mother TPC KO mice for each wave.

According to the post-hoc comparisons, the wave amplitude between the WT and TPC KO mice was different (Figure 3B): The mean amplitude of waves I, III, and IV were significantly higher in the WT (virgin and mother) mice compared to the TPC KO mice (all *p*-values < 0.05). In contrast, the mean amplitude of wave II was significantly smaller in the WT than in the TPC KO mice (*p* < 0.05). Similar effects were also detected at 8 kHz (Supplementary Figure S2).

## 4. Discussion

To summarize, in the virgin TPC KO mice, there was no evidence for threshold alterations compared to the virgin WT mice. In the WT mice, motherhood did not change the acoustic thresholds (as described in Royer et al., 2021 [43]) and the wave amplitudes (even if they tended to display slight increases), but it reduced the latency of the ABR waves (from wave II to wave V). In contrast, for the TPC KO mice, motherhood largely increased the acoustic thresholds at almost all frequencies. In these mice, the latency reduction was less pronounced (but still detected for wave V) and there was no evidence for larger ABR waves in the mothers compared to virgins. Except for wave II (larger in TPC KO mice), the wave amplitudes were smaller in the KO TPC mice (virgin and mother) than in the WT.

As found in the CBA mice using clicks as stimuli [45], we also found that motherhood reduced the response latency in the C57BL/6 WT mice. It is possible that neuromodulators and/or neuro-hormones promoted this effect since neuromodulators impact the response latencies as early as the first relays in the auditory system (cochlear nucleus [46]; inferior colliculus [47,48]). The absence of TPC channels can potentially reduce the neuronal plasticity [37] or the release of neuro-hormones and neuromodulators, which may explain the unchanged latency and amplitude in the TPC KO mothers compared to the virgins.

These results indicate that the absence of TPC channels under normal physiological conditions did not induce a major apparent auditory deficit, even if we observed a response of the auditory nerve on average lower (amplitude of the wave I in ABRs of virgin TPC KO mice), which seems to be compensated for by a larger wave II (i.e., the wave II started higher than that of the WT mice due to the smaller amplitude of wave I). Nevertheless, it appears that in motherhood conditions, the absence of the TPC channels would induce, on average, a reduction in the hearing threshold in the KO mice.

The TPC channels are ubiquitous and seem to play many roles depending on the cell type studied [27,49,50]. Histological observations indicate that TPC channels are present in the endo-lysosomes of cochlear cells (Michalski et al., personal communication), but their roles in these cells have not been investigated yet. We know that the TPC channels are recruited by G coupled-protein receptor agonists such as glutamate and are also involved in the regulation of secretion. Additionally, they have shown to be key components of the autophagy in skeletal cells and cardiomyocytes [33,34,35]. It is therefore possible that they play a similar role in the cochlea. Indeed, autophagy fluxes are involved in the inner ear and cochlea development, and hearing impairments linked to autophagy defects in the cochlea have been observed during normal aging or due to a genetic defect [25]. Several studies have shown that lysosomes are important in cell membrane repair and several lysosomal storage diseases have been associated with cochlear cell degeneration [26]. One example is mucolipidosis IV, where the deletion of the endo-lysosomal channels TRPML1 and 3 led to lysosome dysfunctions and severe hearing deficit [24]. The abrupt physiological change occurring at motherhood associated with an absence of the TPC channel can potentially induce a dysregulation of autophagy in cochlea, which would lead (in the worst scenario) to cell death for the hair cells, a decrease in the number of ribbon synapses, or even a reduction in the number of auditory nerve fibers. These potential alterations could explain the increase in the ABR thresholds of the TPC KO mice but this hypothesis requires further investigations since several other mechanisms could be at play such as structural remodeling, which might depend on hormones or neuromodulators release that are altered in the TPC KO mice. In addition to motherhood, exposure to loud sounds (above or just below the authorized level of 80 dBA during 8 h of work, ISO 1996-1, 2003) can be a way to reveal changes between the WT and TPC KO mice. However, in the case of noise exposure at 80 dBA, it is only with very long lasting noise exposure that effects can be observed [51].

Here, we quantified the ABR thresholds of the WT and TPC KO mother mice at 9–10 days postpartum to be sure of the impact of motherhood on possible audiogram alterations in the TPC KO mice. However, it is important to bear in mind that the majority of human studies on ARHL linked to otosclerosis or sudden hearing loss onset have been analyzed in pregnant women [52,53,54], although some studies have been conducted on postpartum cohorts [55,56]. Thus, it is possible that the increase in thresholds observed in the mother TPC KO mice already exists during pregnancy. In fact, the hormonal changes, which are supposed to partly explain ARHL in pregnant women or women who have had children, already exist from the beginning of pregnancy. Our preliminary ABR data obtained on the TPC KO mother mice during pregnancy (data not shown) suggests that ABR thresholds can also be higher compared to the WT mother mice.

In addition to autophagy fluxes, human studies on ARHL and sudden hearing loss in pregnancy have proposed the dysfunction of various cellular processes in which TPCs may actually have a role. For instance, an otosclerosis link to cochlear osteoclast dysfunction and otosclerosis [57] has also been proposed to underlie the sudden hearing loss in pregnancy and TPC deletion might favor such a pathological process since the TPC has been identified as a regulator of osteoclastogenesis [58]. Finally, the TPCs are ubiquitously expressed, and their deletion alters numerous physiological processes that are linked with several risk factors for ARHL such as hypertension, diabetes, immune, and cardiovascular defects. Indeed, the TPC2 is involved in insulin secretion by the pancreas [28] and in the adrenergic modulation of the cardiac function [35,59] whereas in vivo pharmacological inhibition of the TPC pathway has been reported to increase blood pressure [60]. All of these broad roles of the ubiquitous TPC suggest that the hearing defect observed in these studies could have multiple mechanisms that will need further investigation. Our study provides here some molecular evidence that TPC mutations/deletions that affect numerous physiological functions might be a risk factor for particular physiological processes such as pregnancy.

## Data Availability

The data presented in this study are available upon request from the corresponding author.

## Supplementary Materials

The following supporting information can be downloaded at: https://www.mdpi.com/article/10.3390/biomedicines10071708/s1, Supplementary Figure S1: ABR thresholds at 8 and 16 kHz as a function of the age of the mice. Supplementary Figure S2: Mean latency and mean amplitude of the five ABR waves at 8 kHz at 70 dB.

## References

[1] Bowl M.R., Simon M.M., Ingham N.J., Greenaway S., Santos L., Cater H., Taylor S., Mason J., Kurbatova N., Pearson S. (2017). A Large Scale Hearing Loss Screen Reveals an Extensive Unexplored Genetic Landscape for Auditory Dysfunction. Nat. Commun..

[2] Michalski N., Petit C. (2019). Genes Involved in the Development and Physiology of Both the Peripheral and Central Auditory Systems. Annu. Rev. Neurosci..

[3] Kumar S., Deffenbacher K., Marres H.A.M., Cremers C.W.R.J., Kimberling W.J. (2000). Genomewide Search and Genetic Localization of a Second Gene Associated with Autosomal Dominant Branchio-Oto-Renal Syndrome: Clinical and Genetic Implications. Am. J. Hum. Genet..

[4] Neyroud N., Tesson F., Denjoy I., Leibovici M., Donger C., Barhanin J., Fauré S., Gary F., Coumel P., Petit C. (1997). A Novel Mutation in the Potassium Channel Gene KVLQT1 Causes the Jervell and Lange-Nielsen Cardioauditory Syndrome. Nat. Genet..

[5] Bondurand N., Moal F.D.-L., Stanchina L., Collot N., Baral V., Marlin S., Attie-Bitach T., Giurgea I., Skopinski L., Reardon W. (2007). Deletions at the SOX10 Gene Locus Cause Waardenburg Syndrome Types 2 and 4. Am. J. Hum. Genet..

[6] Elliott K.L., Fritzsch B., Yamoah E.N., Zine A. (2022). Age-Related Hearing Loss: Sensory and Neural Etiology and Their Interdependence. Front. Aging Neurosci..

[7] Moser T., Predoehl F., Starr A. (2013). Review of Hair Cell Synapse Defects in Sensorineural Hearing Impairment. Otol. Neurotol. Off. Publ. Am. Otol. Soc. Am. Neurotol. Soc. Eur. Acad. Otol. Neurotol..

[8] Han C., Someya S. (2013). Mouse Models of Age-Related Mitochondrial Neurosensory Hearing Loss. Mol. Cell. Neurosci..

[9] Keithley E.M. (2020). Pathology and Mechanisms of Cochlear Aging. J. Neurosci. Res..

[10] Tawfik K.O., Klepper K., Saliba J., Friedman R.A. (2020). Advances in Understanding of Presbycusis. J. Neurosci. Res..

[11] Kujawa S.G., Liberman M.C. (2006). Acceleration of Age-Related Hearing Loss by Early Noise Exposure: Evidence of a Misspent Youth. J. Neurosci. Off. J. Soc. Neurosci..

[12] Kujawa S.G., Liberman M.C. (2009). Adding Insult to Injury: Cochlear Nerve Degeneration after “Temporary” Noise-Induced Hearing Loss. J. Neurosci. Off. J. Soc. Neurosci..

[13] Schuknecht H.F., Gacek M.R. (1993). Cochlear Pathology in Presbycusis. Ann. Otol. Rhinol. Laryngol..

[14] Kurata N., Schachern P.A., Paparella M.M., Cureoglu S. (2016). Histopathologic Evaluation of Vascular Findings in the Cochlea in Patients With Presbycusis. JAMA Otolaryngol. Neck Surg..

[15] Schulte B.A., Schmiedt R.A. (1992). Lateral Wall Na, K-ATPase and Endocochlear Potentials Decline with Age in Quiet-Reared Gerbils. Hear. Res..

[16] Gratton M.A., Schmiedt R.A., Schulte B.A. (1996). Age-Related Decreases in Endocochlear Potential Are Associated with Vascular Abnormalities in the Stria Vascularis. Hear. Res..

[17] Gratton M.A., Schulte B.A., Smythe N.M. (1997). Quantification of the Stria Vascularis and Strial Capillary Areas in Quiet-Reared Young and Aged Gerbils. Hear. Res..

[18] Diaz R.C., Vazquez A.E., Dou H., Wei D., Cardell E.L., Lingrel J., Shull G.E., Doyle K.J., Yamoah E.N. (2007). Conservation of Hearing by Simultaneous Mutation of Na,K-ATPase and NKCC1. J. Assoc. Res. Otolaryngol..

[19] Lang H., Jyothi V., Smythe N.M., Dubno J.R., Schulte B.A., Schmiedt R.A. (2010). Chronic Reduction of Endocochlear Potential Reduces Auditory Nerve Activity: Further Confirmation of an Animal Model of Metabolic Presbyacusis. J. Assoc. Res. Otolaryngol..

[20] Schmiedt R.A., Gordon-Salant S., Frisina R.D., Popper A.N., Fay R.R. (2010). The Physiology of Cochlear Presbycusis. The Aging Auditory System.

[21] Smith H.W. (1948). Effect of Pregnancy on Otosclerosis. Arch. Otolaryngol..

[22] Fabbris C., Molteni G., Tommasi N., Marchioni D. (2022). Does Pregnancy Have an Influence on Otosclerosis?. J. Laryngol. Otol..

[23] Frosolini A., Marioni G., Gallo C., de Filippis C., Lovato A. (2021). Audio-Vestibular Disorders and Pregnancy: A Systematic Review. Am. J. Otolaryngol..

[24] Wiwatpanit T., Remis N.N., Ahmad A., Zhou Y., Clancy J.C., Cheatham M.A., García-Añoveros J. (2018). Codeficiency of Lysosomal Mucolipins 3 and 1 in Cochlear Hair Cells Diminishes Outer Hair Cell Longevity and Accelerates Age-Related Hearing Loss. J. Neurosci. Off. J. Soc. Neurosci..

[25] Magariños M., Pulido S., Aburto M.R., de Iriarte Rodríguez R., Varela-Nieto I. (2017). Autophagy in the Vertebrate Inner Ear. Front. Cell Dev. Biol..

[26] Giffen K.P., Li Y., Liu H., Zhao X.-C., Zhang C.-J., Shen R.-J., Wang T., Janesick A., Chen B.-B., Gong S.-S. (2022). Mutation of SLC7A14 Causes Auditory Neuropathy and Retinitis Pigmentosa Mediated by Lysosomal Dysfunction. Sci. Adv..

[27] Martucci L.L., Cancela J.-M. (2022). Neurophysiological Functions and Pharmacological Tools of Acidic and Non-Acidic Ca^2+^ Stores. Cell Calcium.

[28] Calcraft P.J., Ruas M., Pan Z., Cheng X., Arredouani A., Hao X., Tang J., Rietdorf K., Teboul L., Chuang K.-T. (2009). NAADP Mobilizes Calcium from Acidic Organelles through Two-Pore Channels. Nature.

[29] Brailoiu E., Hooper R., Cai X., Brailoiu G.C., Keebler M.V., Dun N.J., Marchant J.S., Patel S. (2010). An Ancestral Deuterostome Family of Two-Pore Channels Mediates Nicotinic Acid Adenine Dinucleotide Phosphate-Dependent Calcium Release from Acidic Organelles. J. Biol. Chem..

[30] Zong X., Schieder M., Cuny H., Fenske S., Gruner C., Rötzer K., Griesbeck O., Harz H., Biel M., Wahl-Schott C. (2009). The Two-Pore Channel TPCN2 Mediates NAADP-Dependent Ca(^2+^)-Release from Lysosomal Stores. Pflugers Arch..

[31] Pitt S.J., Lam A.K.M., Rietdorf K., Galione A., Sitsapesan R. (2014). Reconstituted Human TPC1 Is a Proton-Permeable Ion Channel and Is Activated by NAADP or Ca^2+^. Sci. Signal..

[32] Gerndt S., Chen C.-C., Chao Y.-K., Yuan Y., Burgstaller S., Scotto Rosato A., Krogsaeter E., Urban N., Jacob K., Nguyen O.N.P. (2020). Agonist-Mediated Switching of Ion Selectivity in TPC2 Differentially Promotes Lysosomal Function. eLife.

[33] Cang C., Zhou Y., Navarro B., Seo Y.-J., Aranda K., Shi L., Battaglia-Hsu S., Nissim I., Clapham D.E., Ren D. (2013). MTOR Regulates Lysosomal ATP-Sensitive Two-Pore Na(^+^) Channels to Adapt to Metabolic State. Cell.

[34] Lin P.-H., Duann P., Komazaki S., Park K.H., Li H., Sun M., Sermersheim M., Gumpper K., Parrington J., Galione A. (2015). Lysosomal Two-Pore Channel Subtype 2 (TPC2) Regulates Skeletal Muscle Autophagic Signaling. J. Biol. Chem..

[35] García-Rúa V., Feijóo-Bandín S., Rodríguez-Penas D., Mosquera-Leal A., Abu-Assi E., Beiras A., María Seoane L., Lear P., Parrington J., Portolés M. (2016). Endolysosomal Two-Pore Channels Regulate Autophagy in Cardiomyocytes. J. Physiol..

[36] Gerasimenko J.V., Charlesworth R.M., Sherwood M.W., Ferdek P.E., Mikoshiba K., Parrington J., Petersen O.H., Gerasimenko O.V. (2015). Both RyRs and TPCs Are Required for NAADP-Induced Intracellular Ca^2+^ Release. Cell Calcium.

[37] Foster W.J., Taylor H.B.C., Padamsey Z., Jeans A.F., Galione A., Emptage N.J. (2018). Hippocampal MGluR1-Dependent Long-Term Potentiation Requires NAADP-Mediated Acidic Store Ca^2+^ Signaling. Sci. Signal..

[38] Favia A., Desideri M., Gambara G., D’Alessio A., Ruas M., Esposito B., Del Bufalo D., Parrington J., Ziparo E., Palombi F. (2014). VEGF-Induced Neoangiogenesis Is Mediated by NAADP and Two-Pore Channel-2-Dependent Ca^2+^ Signaling. Proc. Natl. Acad. Sci. USA.

[39] Ruas M., Galione A., Parrington J. (2015). Two-Pore Channels: Lessons from Mutant Mouse Models. Messenger.

[40] Chabout J., Cressant A., Hu X., Edeline J.-M., Granon S. (2013). Making Choice between Competing Rewards in Uncertain vs. Safe Social Environment: Role of Neuronal Nicotinic Receptors of Acetylcholine. Front. Hum. Neurosci..

[41] Chaussenot R., Edeline J.-M., Le Bec B., El Massioui N., Laroche S., Vaillend C. (2015). Cognitive Dysfunction in the Dystrophin-Deficient Mouse Model of Duchenne Muscular Dystrophy: A Reappraisal from Sensory to Executive Processes. Neurobiol. Learn. Mem..

[42] Martucci L.L., Amar M., Chaussenot R., Benet G., Bauer O., de Zélicourt A., Nosjean A., Launay J.-M., Callebert J., Sebrié C. (2019). A Multiscale Analysis in CD38-/- Mice Unveils Major Prefrontal Cortex Dysfunctions. FASEB J. Off. Publ. Fed. Am. Soc. Exp. Biol..

[43] Royer J., Huetz C., Occelli F., Cancela J.-M., Edeline J.-M. (2021). Enhanced Discriminative Abilities of Auditory Cortex Neurons for Pup Calls Despite Reduced Evoked Responses in C57BL/6 Mother Mice. Neuroscience.

[44] Zheng Q.Y., Johnson K.R., Erway L.C. (1999). Assessment of Hearing in 80 Inbred Strains of Mice by ABR Threshold Analyses. Hear. Res..

[45] Miranda J.A., Shepard K.N., McClintock S.K., Liu R.C. (2014). Adult Plasticity in the Subcortical Auditory Pathway of the Maternal Mouse. PLoS ONE.

[46] Kössl M., Vater M. (1989). Noradrenaline Enhances Temporal Auditory Contrast and Neuronal Timing Precision in the Cochlear Nucleus of the Mustached Bat. J. Neurosci. Off. J. Soc. Neurosci..

[47] Hurley L.M., Pollak G.D. (1999). Serotonin Differentially Modulates Responses to Tones and Frequency-Modulated Sweeps in the Inferior Colliculus. J. Neurosci. Off. J. Soc. Neurosci..

[48] Hurley L.M., Pollak G.D. (2005). Serotonin Shifts First-Spike Latencies of Inferior Colliculus Neurons. J. Neurosci. Off. J. Soc. Neurosci..

[49] Davis L.C., Morgan A.J., Galione A. (2022). Acidic Ca^2+^ Stores and Immune-Cell Function. Cell Calcium.

[50] Marchant J.S., Patel S. (2015). Two-Pore Channels at the Intersection of Endolysosomal Membrane Traffic. Biochem. Soc. Trans..

[51] Occelli F., Hasselmann F., Bourien J., Puel J.-L., Desvignes N., Wiszniowski B., Edeline J.-M., Gourévitch B. (2022). Temporal Alterations to Central Auditory Processing without Synaptopathy after Lifetime Exposure to Environmental Noise. Cereb. Cortex.

[52] Zhang B.-Y., Young Y.-H. (2017). Sudden Deafness during Antepartum versus Postpartum Periods. ORL J. Oto-Rhino-Laryngol. Its Relat. Spec..

[53] Xu M., Jiang Q., Tang H. (2019). Sudden Sensorineural Hearing Loss during Pregnancy: Clinical Characteristics, Management and Outcome. Acta Otolaryngol..

[54] Lyu Y.-L., Zeng F.-Q., Zhou Z., Yan M., Zhang W., Liu M., Ke Z.-Y. (2020). Intratympanic Dexamethasone Injection for Sudden Sensorineural Hearing Loss in Pregnancy. World J. Clin. Cases.

[55] Crompton M., Cadge B.A., Ziff J.L., Mowat A.J., Nash R., Lavy J.A., Powell H.R.F., Aldren C.P., Saeed S.R., Dawson S.J. (2019). The Epidemiology of Otosclerosis in a British Cohort. Otol. Neurotol. Off. Publ. Am. Otol. Soc. Am. Neurotol. Soc. Eur. Acad. Otol. Neurotol..

[56] Lippy W.H., Berenholz L.P., Schuring A.G., Burkey J.M. (2005). Does Pregnancy Affect Otosclerosis?. Laryngoscope.

[57] Xu K., Bai X., Chen S., Xie L., Qiu Y., Li H., Sun Y. (2021). CCDC_154_ Mutant Caused Abnormal Remodeling of the Otic Capsule and Hearing Loss in Mice. Front. Cell Dev. Biol..

[58] Notomi T., Ezura Y., Noda M. (2012). Identification of Two-Pore Channel 2 as a Novel Regulator of Osteoclastogenesis. J. Biol. Chem..

[59] Capel R.A., Bolton E.L., Lin W.K., Aston D., Wang Y., Liu W., Wang X., Burton R.-A.B., Bloor-Young D., Shade K.-T. (2015). Two-Pore Channels (TPC2s) and Nicotinic Acid Adenine Dinucleotide Phosphate (NAADP) at Lysosomal-Sarcoplasmic Reticular Junctions Contribute to Acute and Chronic β-Adrenoceptor Signaling in the Heart. J. Biol. Chem..

[60] Favia A., Pafumi I., Desideri M., Padula F., Montesano C., Passeri D., Nicoletti C., Orlandi A., Del Bufalo D., Sergi M. (2016). NAADP-Dependent Ca^2+^ Signaling Controls Melanoma Progression, Metastatic Dissemination and Neoangiogenesis. Sci. Rep..

