# Supplementary material for: Unexpected Motherhood-Triggered Hearing Loss in the Two-Pore Channel (TPC) Mutant Mouse
Source: Biomedicines. 2022 Jul 15;10(7):1708. doi: 10.3390/biomedicines10071708 (PMC9312904; doi:10.3390/biomedicines10071708)
Supplement: Supplementary file 1 [file biomedicines-10-01708-s001.zip › biomedicines-1774554-supplementary.pdf]

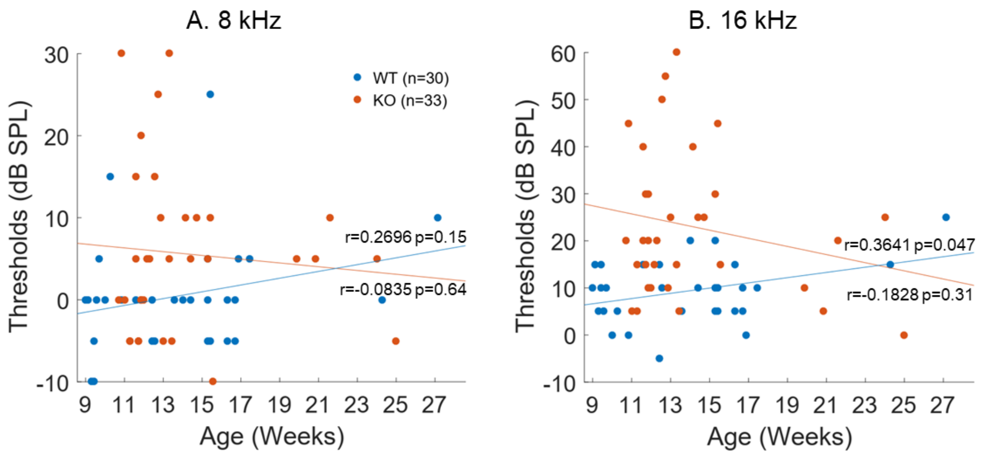

Supplementary Figure S1. ABR thresholds at 8 and 16 kHz as a function of the age of the mice.
A: ABR thresholds at 8 kHz of WT and TPC KO mice (virgins and mothers grouped) as a function of their age (in weeks). Threshold was not related to the age of the WT and TPC KO mice. The higher thresholds in TPC KO mice were obtained for young mother mice (between 11 and 13 weeks).
B: ABR thresholds at 16 kHz of WT and TPC KO mice (virgins and mothers mingled) compared to their age (in weeks). Threshold was slightly related to the age in the WT (p=0.047) but not in TPC KO mice (p=0.31). The higher thresholds in TPC KO mice were obtained in young mother mice (between 11 and 13 weeks).

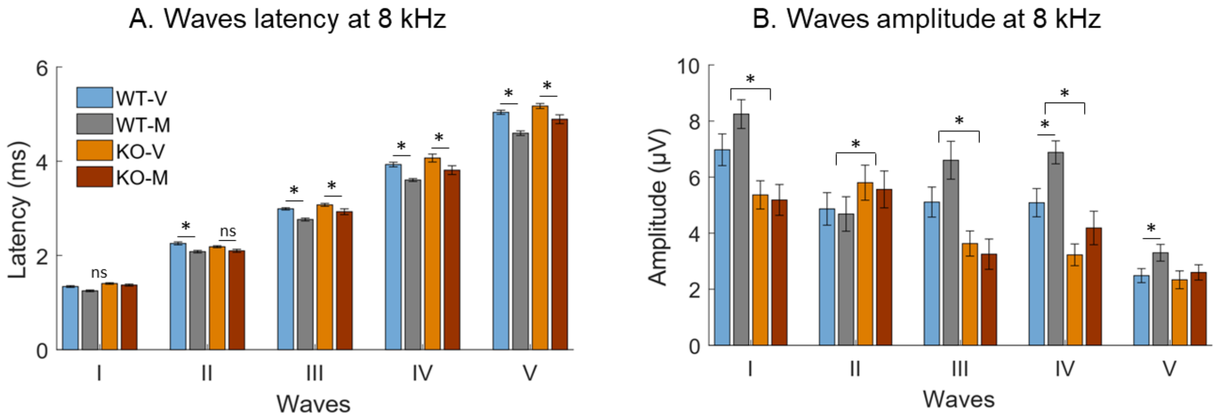

Supplementary Figure S2. Mean latency and mean amplitude of the five ABR waves at 8 kHz at 70dB.

A: Latency distribution of waves I to V for each group at 8 kHz. The latencies of waves II to V were significantly shorter in WT mothers compared to WT virgins. In TPC KO mother mice the latencies of waves III to V were significantly shorter compared to TPC KO virgins.

B: Amplitude distribution of waves I to V for each group at 8 kHz. The amplitudes of waves were not different between virgin and mother mice (WT and TPC KO), except for wave IV and V between WT virgins and WT mothers. The wave amplitude between WT and TPC KO mice also differed for four of the fives ABR waves.
